# Interpretable Diagnosis of Pulmonary Emphysema on Low-Dose CT Using ResNet Embeddings

**DOI:** 10.3390/jimaging12010051

**Published:** 2026-01-21

**Authors:** Talshyn Sarsembayeva, Madina Mansurova, Ainash Oshibayeva, Stepan Serebryakov

**Affiliations:** 1 Faculty of Information Technologies and Artificial Intelligence, Department of Artificial Intelligence and Big Data, Al-Farabi Kazakh National University, Almaty 050040, Kazakhstan; madina.mansurova@kaznu.edu.kz (M.M.); serebryakov0906@gmail.com (S.S.); 2 Faculty of Medicine, Department of Public Health and Scientific Research, Khoja Akhmet Yassawi International Kazakh-Turkish University, Turkistan 161200, Kazakhstan; 3 Smart Parking Technologies Ltd., Almaty 010000, Kazakhstan

**Keywords:** emphysema, low-dose CT, deep learning, ResNet, feature embeddings, explainable AI, weak supervision

## Abstract

Accurate and interpretable detection of pulmonary emphysema on low-dose computed tomography (LDCT) remains a critical challenge for large-scale screening and population health studies. This work proposes a quality-controlled and interpretable deep learning pipeline for emphysema assessment using ResNet-152 embeddings. The pipeline integrates automated lung segmentation, quality-control filtering, and extraction of 2048-dimensional embeddings from mid-lung patches, followed by analysis using logistic regression, LASSO, and recursive feature elimination (RFE). The embeddings are further fused with quantitative CT (QCT) markers, including %LAA, Perc15, and total lung volume (TLV), to enhance robustness and interpretability. Bootstrapped validation demonstrates strong diagnostic performance (ROC-AUC = 0.996, PR-AUC = 0.962, balanced accuracy = 0.931) with low computational cost. The proposed approach shows that ResNet embeddings pretrained on CT data can be effectively reused without retraining for emphysema characterization, providing a reproducible and explainable framework suitable as a research and screening-support framework for population-level LDCT analysis.

## 1. Introduction

Chronic obstructive pulmonary disease (COPD) remains one of the leading causes of morbidity and mortality worldwide. According to the World Health Organization, it ranks among the top four causes of death globally and is projected to increase in prevalence in the coming decades. The situation is particularly alarming in low- and middle-income countries (LMICs), where up to 90% of all COPD-related deaths occur among people under 70 years of age. In addition to high mortality, COPD significantly contributes to the global disease burden, ranking eighth among causes of ill health measured in disability-adjusted life years (DALYs). The main risk factors for the development of COPD include tobacco smoking and indoor air pollution. Smoking is responsible for over 70% of COPD cases in high-income countries, while household air pollution is the main cause in LMICs, where tobacco-related COPD accounts for 30–40% of cases [1]. Pulmonary emphysema, which causes hyperinflation and poor gas exchange due to irreversible destruction of alveolar walls and a loss of lung tissue elasticity, is one of the main morphological phenotypes of COPD [2].

Although spirometry-based indices such as FEV1 and the FEV1/FVC ratio remain the clinical standard for diagnosing chronic obstructive pulmonary disease (COPD) [3], computed tomography (CT) provides direct insight into structural alterations of the lung parenchyma, most notably emphysematous destruction [4]. In CT-based quantitative analysis, emphysema is commonly characterized using the low attenuation area percentage (LAA%), which represents the fraction of lung voxels with attenuation values below −950 Hounsfield Units (HU) [5].

The increasing adoption of low-dose CT (LDCT) in lung cancer screening programs [6] has substantially expanded the availability of volumetric chest imaging. Beyond oncological assessment, these scans enable opportunistic evaluation of smoking-related lung abnormalities, including emphysema, without additional imaging burden.

Deep learning approaches, and convolutional neural networks (CNNs) in particular, have achieved strong results in medical image analysis tasks, including the detection of pulmonary diseases [7]. Despite their predictive performance, many CNN-based systems function as opaque models, offering limited insight into the rationale behind their decisions. This lack of transparency remains a major barrier to clinical adoption, motivating growing interest in explainable artificial intelligence (XAI) within healthcare applications [8].

In this work, we present an interpretable framework for emphysema detection from low-dose CT scans that leverages deep feature embeddings extracted from a pretrained ResNet152 architecture [9]. Rather than training an end-to-end deep classifier, we utilize the 2048-dimensional representation obtained from the global average pooling layer and apply conventional statistical models, including logistic and linear regression. This design choice prioritizes reproducibility, computational efficiency, and transparency of model behavior.

The study is guided by the following objectives:

- To assess whether deep feature embeddings encode sufficient information for reliable emphysema classification;
- To evaluate whether a reduced subset of features can preserve diagnostic performance while improving interpretability;
- To establish a regression-based approach for estimating emphysema severity, using LAA% as a continuous outcome variable.

Our approach offers a transparent and adaptable tool for automated emphysema diagnosis and can be integrated into existing LDCT analysis pipelines for screening and clinical evaluation.

Our contributions are: (1) a quality-controlled, threshold-justified auto-labeling of emphysema on LDCT; (2) a transparent classifier on top of pretrained CNN embeddings with feature-level attribution; (3) a fusion of deep features with QCT markers (LAA-950, Perc15, TLV) yielding strong performance; (4) rigorous uncertainty quantification (bootstrap CI) and bias control (patient-level splits, class weighting). Unlike previous studies that primarily visualized embeddings using t-SNE or UMAP, our contribution focuses on the design of a fully reproducible, interpretable pipeline with controlled thresholds and bias-aware evaluation.

## 2. Related Work

Automatic detection and quantification of pulmonary emphysema on chest CT has been actively researched for over two decades. Early studies relied on handcrafted radiomic features, such as attenuation histograms, texture descriptors, and spatial patterns of low-density regions [4,5,10]. The LAA% metric, representing the proportion of voxels below −950 HU, remains a widely accepted biomarker.

With the rise of deep learning, CNNs have outperformed traditional methods in diagnosing lung diseases [11,12,13]. COPDNet [11], a ResNet-based framework, demonstrated competitive performance for emphysema-related analysis on low-dose CT, achieving an AUC of 0.886 and outperforming conventional LAA-based measures. In a subsequent large-scale study, Tang et al. [14] trained deep residual networks on LDCT data for population-level COPD case finding. Their model achieved consistently high sensitivity and specificity across both internal and external cohorts, indicating that ResNet architectures are suitable for screening-oriented pulmonary analysis. These works established an important foundation for later studies employing deep residual networks in COPD and emphysema assessment.

Despite their strong performance, deep learning models are often criticized for limited interpretability, which restricts their clinical applicability. To mitigate this limitation, a growing body of research has focused on explainable artificial intelligence (XAI) techniques [8,15]. Methods based on spatial attention, such as class activation mapping (CAM) [16] and Grad-CAM [17], as well as weakly supervised localization approaches [18], provide visual explanations of model focus regions. However, these techniques primarily highlight salient image areas and offer limited insight into the structure of the learned feature representations.

An alternative direction involves analyzing deep feature spaces using dimensionality reduction methods such as principal component analysis (PCA) and t-distributed stochastic neighbor embedding (t-SNE) [19,20]. For example, Wang et al. [20] applied t-SNE to CT-derived deep features and demonstrated partial class separation between pathological and non-pathological cases. Related work by Rajpurkar et al. [21] showed that self-supervised representations can achieve performance comparable to expert radiologists in chest imaging tasks, further highlighting the diagnostic value of learned embeddings.

Hybrid approaches that combine deep feature extraction with interpretable statistical models, such as logistic regression, have been proposed to balance predictive performance and transparency [22]. These frameworks are particularly attractive in medical imaging, where reproducibility, interpretability, and regulatory considerations are critical [23,24].

Building on this line of research, the present study employs feature embeddings extracted from a pretrained ResNet152 model, applies statistical feature selection, and constructs interpretable classification and regression models tailored for emphysema screening in low-dose CT data.

## 3. Materials and Methods

### 3.1. Data

This study utilized the publicly available LIDC-IDRI dataset [25], which provides low-dose chest CT scans in DICOM format. Although the dataset was originally assembled for lung nodule analysis, its volumetric LDCT acquisitions enable secondary investigation of parenchymal lung abnormalities, including emphysema, following appropriate preprocessing and filtering. LIDC-IDRI comprises 1018 LDCT examinations collected through a multi-institutional collaboration involving academic centers and imaging vendors, coordinated under the supervision of U.S. federal agencies (NCI, FNIH, FDA). Each scan is accompanied by structured XML annotations generated by four board-certified thoracic radiologists using a two-stage review process. In the first stage, radiologists independently annotated imaging findings; in the second stage, annotations were revised after reviewing anonymized peer assessments. While the primary focus of these annotations is pulmonary nodules, the dataset also provides sufficient image quality and coverage for quantitative lung analysis.

Earlier versions of the LIDC collection have previously been explored for emphysema-related assessment. For example, Wiemker et al. [26] analyzed LDCT scans from a prototype LIDC database and demonstrated that histogram-based density metrics could capture emphysema-related patterns, supporting the feasibility of quantitative parenchymal analysis using this data source. Of the 1018 available LIDC-IDRI cases, 497 scans were successfully processed through the complete preprocessing pipeline and included in the present study. Exclusions prior to this stage were due to unreadable image series, missing metadata, or technical preprocessing constraints. No cases were excluded solely on the basis of lung segmentation quality.

### 3.2. Proposed Approach

#### 3.2.1. Problem Statement

Emphysema represents a structural component of chronic obstructive pulmonary disease (COPD) and is characterized by irreversible enlargement of distal airspaces together with destruction of alveolar walls. These changes impair gas exchange and lung mechanics and are often difficult to identify at early stages, when imaging findings remain subtle and subject to inter-reader variability.

Computed tomography (CT) provides direct visualization of emphysematous alterations and is therefore central to imaging-based assessment. In routine practice, however, evaluation frequently relies on visual inspection or simple density-based indicators such as the low attenuation area percentage (LAA%). Although LAA% reflects overall disease burden, it may overlook spatially heterogeneous or region-specific abnormalities that do not exceed predefined Hounsfield Unit thresholds.

Progress in deep learning has substantially improved performance in medical image classification tasks. Convolutional neural networks (CNNs), especially when pretrained on large-scale datasets, can capture complex image patterns. Nevertheless, their application to emphysema analysis is constrained by limited availability of annotated clinical data and by the limited interpretability of model outputs, which poses challenges for clinical acceptance and regulatory use.

**Figure 1:**
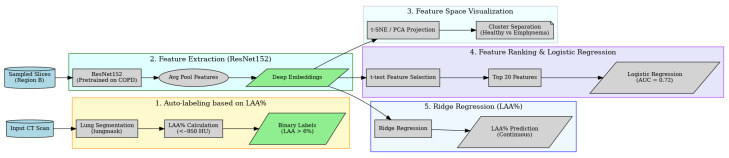
Overview of the explainable emphysema analysis pipeline. The process includes lung segmentation and automatic labeling based on low attenuation area percentage (LAA%), deep feature extraction with ResNet152, visualization with t-SNE/PCA, and interpretation using logistic and ridge regression.

In this work, we develop a compact diagnostic pipeline that combines pretrained CNN embeddings with interpretable feature selection and conventional machine learning classifiers to distinguish emphysematous from non-emphysematous low-dose CT scans. The emphasis is placed on transparency, robustness across patients, and reduced reliance on manual annotation, making the approach suitable for large-scale screening-oriented analyses. We consider the problem of binary classification of chest LDCT scans into emphysematous (y=1) and non-emphysematous (y=0) categories based on a feature vector $x\in R^{d}$ derived from CNN embeddings. The goal is to learn a mapping $f:R^{d}\to \left\{0,1\right\}$ that generalizes well and remains interpretable, where *d* is the dimension of the feature space [14]. An overview is shown in Figure 1.

A detailed overview of the file-based interaction between scripts is presented in the supplementary sequence diagram (see Appendix A).

#### 3.2.2. Pipeline Overview

For each patient, the following preprocessing pipeline was applied:

1. Series Selection: From all available DICOM series, we selected the one with the largest number of slices, assuming it to be the most complete volumetric scan.
2. Slice Loading: The images were sorted by the Z-coordinate and converted into a 3D array with voxel intensities transformed to Hounsfield Units (HU). ImageNet-pretrained ResNet152 embeddings demonstrated strong discriminative capacity when applied to lung-masked LDCT data. LIDC-IDRI was not used during pretraining, and the backbone remained frozen during downstream training to avoid leakage.
3. Density-Based Filtering: HU values were clipped to [−1000, 400] for normalization and visualization purposes only. No scans were excluded based on HU thresholds; quality control relied on lung segmentation integrity and radiological plausibility checks. Quality control relied on lung-segmentation checks and radiological plausibility.
4. Automatic Annotation:
  - All volumes were resampled to isotropic resolution of 1×1×1 mm^3^ to ensure inter-patient comparability.
  - Lung segmentation was performed using the lungmask tool (trunk U-Net model trained on VESSEL12).
  - Low Attenuation Area (LAA%):
    – Voxels with HU below −950 were considered potential emphysematous regions.
    – LAA% was calculated as the proportion of such voxels within the segmented lung mask.
    – Patients with LAA% ≥ 6% and mean lung density below −850 HU were labeled as positive (label = 1); the rest were considered negative (label = 0 These criteria were used solely for label assignment and were not used as direct inputs for the embedding-only classification models. ).
5. Auxiliary Data Saved:
  - Segmentation masks.
  - Slice visualizations with overlaid masks.
  - LAA distribution curves per axial slice.

In total, 497 LDCT scans were processed. Of these:

- 45 cases (9%) were labeled as positive (evidence of emphysema).
- 452 cases (91%) were labeled as negative.

Such class imbalance is typical in screening cohorts. To enable controlled exploratory analyses under balanced class conditions, a dedicated subset was assembled. This subset comprised all emphysema-positive cases and an equal number of randomly selected negative scans. For each selected case, the corresponding data files (including .npy volumes and associated .json spacing metadata) were collected into a separate directory, along with a label file containing the filtered patient annotations.

**Figure 2:**
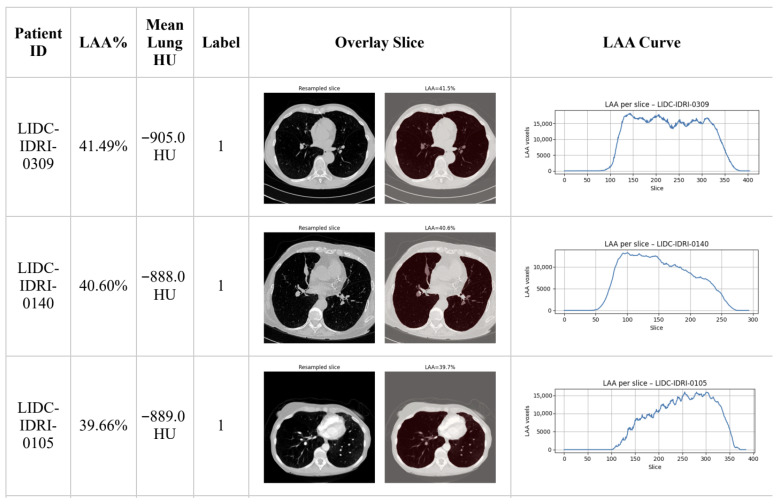
Examples of CT-based emphysema labeling using the proposed pipeline. The table shows LAA percentage, mean lung density, representative slices with segmentation overlays, and the LAA distribution curve across axial slices. Detailed patient-level analysis is provided in Appendix A.

The outcome of the automatic labeling procedure is illustrated in Figure 2, where three representative cases are shown. For each patient, the computed LAA% and mean lung density are presented alongside axial LDCT slices with lung segmentation masks and the corresponding LAA distribution curve. All presented cases satisfy the positive class condition based on LAA% ≥ 6% and average HU below −850.

To address class imbalance, we trained on the full cohort with inverse-frequency class weights and patient-level stratification (70/15/15). Patient IDs are logged to ensure no cross-split leakage. To address class imbalance, all primary models were trained on the full cohort using inverse-frequency class weighting with patient-level stratified splits (70/15/15). Balanced subsets created by downsampling the majority class were used exclusively for probing analyses and representation diagnostics, and not for final performance evaluation; details in Table 1.

To further characterize the distribution of emphysema severity within the full dataset, we computed descriptive statistics of the LAA% across all 497 LDCT volumes. As shown in Table 1, the average LAA% was 3.31%, with a standard deviation of 6.22%. The majority of cases exhibited low emphysema burden, with a median of only 0.73%, while the most severe cases reached up to 41.49%.

Additionally, Table 2 presents the ten patients with the highest LAA% values. Most of them were assigned to the positive class according to our labeling criteria (LAA% ≥ 6% and mean HU < −850), supporting the validity of the automatic labeling strategy. Notably, some scans with high LAA% values did not meet the combined labeling criteria due to higher mean lung attenuation, illustrating the role of the auxiliary HU constraint in preventing false-positive labeling driven by volume or noise effects.

These statistics illustrate the expected class imbalance in the original cohort and provide quantitative insight into the spectrum of emphysema severity, further justifying the construction of a balanced subset for downstream classification.

### 3.3. Comparison of Dimensionality Reduction Techniques

**Figure 3:**
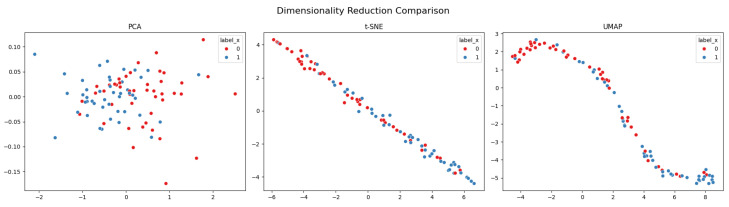
Comparison of dimensionality reduction techniques applied to ResNet152 embeddings. Each dot represents a patient sample colored by ground truth label (red = non-emphysematous, blue = emphysematous).

To further interpret the learned feature space, we applied three commonly used dimensionality reduction techniques—Principal Component Analysis (PCA), t-Distributed Stochastic Neighbor Embedding (t-SNE), and Uniform Manifold Approximation and Projection (UMAP)—to the CNN embeddings (2048-dimensional vectors extracted from the global average pooling layer of ResNet152). The comparison is shown in Figure 3.

We compared three dimensionality reduction methods: PCA [27], t-SNE [28], and UMAP [29]. While PCA provides a linear projection based on global variance, t-SNE and UMAP aim to preserve neighborhood structure in a nonlinear embedding space. The PCA plot shows poor separation between the two classes, suggesting that the feature space is not linearly separable. t-SNE and UMAP reveal more structure, with UMAP in particular capturing a smooth manifold where emphysematous and non-emphysematous samples are arranged along a continuum. The visualization suggests that the CNN-derived embeddings capture continuous variation in lung parenchymal patterns rather than forming strictly separated clusters. This behavior indicates that the learned representation reflects gradual pathological changes and may therefore be suitable for downstream analyses such as severity regression or subtype exploration.

To further characterize the structure of the embedding space, we computed standard unsupervised separation metrics. The silhouette score was 0.235, the Calinski–Harabasz index reached 203.4, and the Davies–Bouldin index was 0.82. Together, these values indicate a modest but non-random separation between emphysema-positive and -negative samples. While the embeddings do not form clearly distinct clusters, the metrics point to the presence of discriminative structure, which aligns with the observed classification results and suggests room for improvement through task-specific supervision or refined representation learning.

### 3.4. Data Preprocessing and Threshold Justification

**Figure 4:**
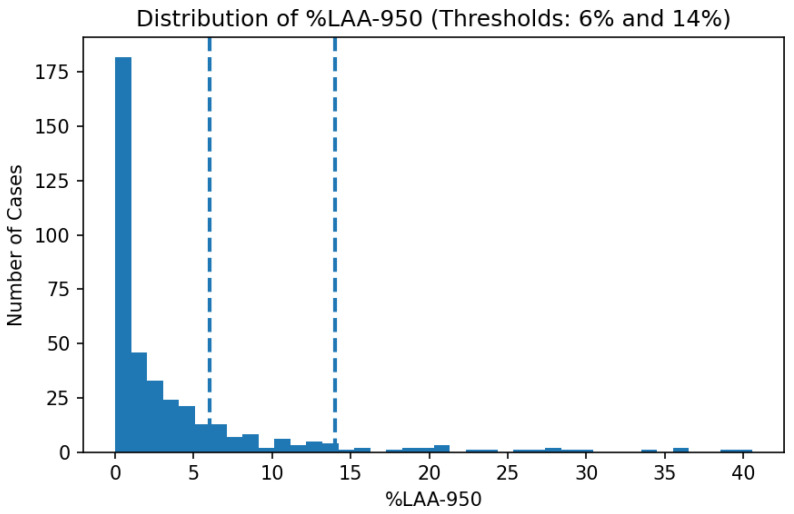
Distribution of %LAA-950 across the LIDC-IDRI cohort. Dotted lines indicate the 6% (diagnostic) and 14% (severe) thresholds.

**Figure 5:**
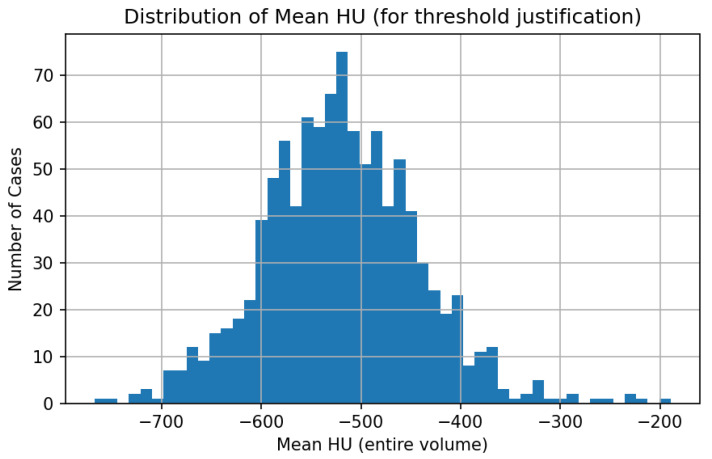
Scatterplot of %LAA-950 vs. mean lung HU. Negative correlation indicates greater emphysema in lower-density lungs.

**Figure 6:**
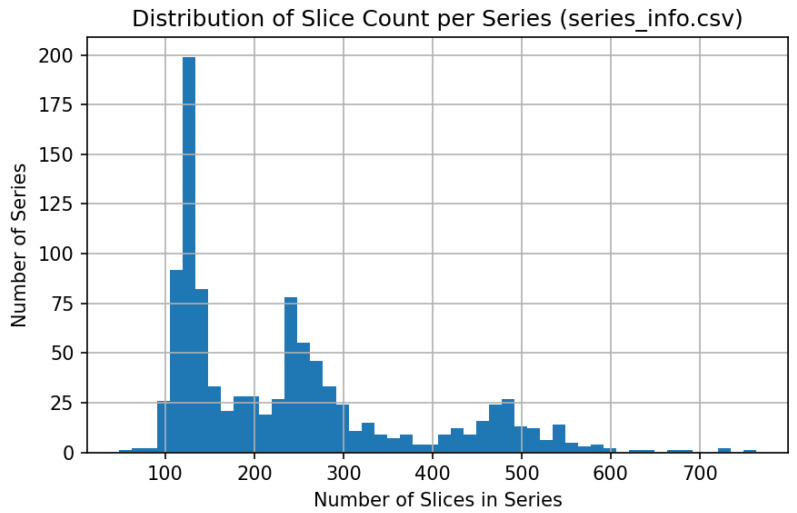
Distribution of slice count per series.

All CT volumes were resampled to an isotropic voxel spacing of 1 mm^3^ prior to quantitative analysis, in line with common QCT practice. Lung segmentation was performed using the publicly available LungMask model trained on VESSEL12 [30,31]. Quantitative emphysema was assessed using the standard %LAA-950 metric—the proportion of voxels with HU below −950 within the lung mask. A threshold of 6% was applied to assign emphysema-positive labels, as supported by Occhipinti et al. [32]. A more severe cut-off of 14% was also marked in figures. Mean lung attenuation (HU), total lung volume (TLV), and Perc15 (the 15th percentile of voxel intensities) were extracted for each scan and used as additional quality control (QC) and fusion features. Previous studies indicate that TLV strongly affects %LAA due to inspiration effort [33], and that denoising or reconstruction kernel impacts were considered in interpretation [34]. The full histogram of %LAA values, mean HU and slice count per scan is shown in Figure 4, Figure 5 and Figure 6.

All scans were stratified into training, validation, and test sets using a 70/15/15 split ratio, preserving class distribution. Table 3 summarizes the key metrics for each subset, showing that balance and radiological metrics were consistent across splits. The low-dose CT cohort remained representative in terms of emphysema severity, lung volume, and parenchymal density. Patient identifiers were monitored to prevent overlap between data splits.

### 3.5. Label Definition and Quantitative CT Criteria

Emphysema labels were assigned based on two quantitative CT measures: the proportion of LAA% and the average lung attenuation. A threshold of LAA ≥ 6% was selected as an operational cutoff consistent with commonly used ranges in emphysema imaging studies, where values between approximately 5–10% are associated with clinically relevant parenchymal destruction.

Mean lung attenuation < −850 HU was used as an auxiliary constraint to ensure physiologically plausible lung density values. Analysis of the empirical distribution of mean lung attenuation across the cohort demonstrates that this threshold lies within the central mass of observed values rather than at distribution extremes, thereby avoiding label definitions driven by outliers.

Given the limited cohort size, alternative labeling thresholds were not used for full model retraining. The selected criteria therefore represent a dataset-specific operational definition rather than a universal clinical standard.

### 3.6. Lung Segmentation and Quality Control

All lung segmentation masks were subjected to systematic visual quality control. No cases with complete segmentation failure were identified, and therefore no cases were excluded on the basis of segmentation quality.

As voxel-level ground-truth lung masks are not available for the LIDC-IDRI dataset, quantitative overlap metrics such as Dice or Intersection-over-Union could not be computed. Minor boundary inaccuracies were occasionally observed near the mediastinum or diaphragm; however, these did not affect global lung density measurements or LAA-based computations.

### 3.7. Data Splitting and Class Imbalance Handling

The dataset exhibits a pronounced class imbalance, reflecting the natural prevalence of emphysema-related patterns. To account for class imbalance while preserving methodological clarity, two complementary experimental setups were defined.

A balanced subset was first created by randomly downsampling the majority class. This subset was used solely for probing analyses and examination of representation behavior, where equal class sizes support controlled interpretation. Importantly, no final performance estimates were derived from this balanced configuration.

All primary performance results were instead obtained using the full cohort. In this setting, patient-level splits into training, validation, and test sets were applied while maintaining the original class distribution. This strategy reduces the risk of optimistic bias associated with majority-class downsampling and ensures that reported metrics better reflect screening-like prevalence conditions.

### 3.8. Deep Feature Extraction Using ResNet-152

Deep features were extracted using a ResNet-152 convolutional neural network initialized with ImageNet-pretrained weights. The network was used strictly as a fixed feature extractor, and no fine-tuning of its convolutional layers was performed.

Before feature extraction, CT volumes were resampled to isotropic voxel spacing, intensity-normalized, and masked with the corresponding lung segmentation to remove non-pulmonary regions. The resulting data were then processed as two-dimensional axial slices.

Patch sampling was performed within the lung region to ensure that extracted features corresponded to pulmonary parenchyma. Each patch was resized to a spatial resolution compatible with the ResNet-152 input. Deep features were extracted from the penultimate global average pooling layer, resulting in a 2048-dimensional embedding vector per patch.

For each scan, patch-level embeddings were aggregated to obtain a scan-level representation used in subsequent classification and analysis.

### 3.9. Statistical Analysis

Model performance was evaluated on the held-out test set. Uncertainty was quantified using patient-level bootstrap resampling to preserve independence between samples. A fixed number of bootstrap resamples was drawn with replacement from the test cohort, and 95% confidence intervals were estimated using the percentile method.

Given the limited cohort size and the exploratory scope of the present study, no formal hypothesis testing between models (e.g., DeLong tests or bootstrap-based *p*-values) was performed. Model comparisons are therefore presented descriptively using point estimates and confidence intervals.

## 4. Results

### 4.1. Classification Performance

Differences in reported performance metrics reflect distinct model configurations: logits-based and embedding-only probes yield moderate discrimination, whereas the near-perfect performance is observed only for the multimodal fusion model.

**Figure 7:**
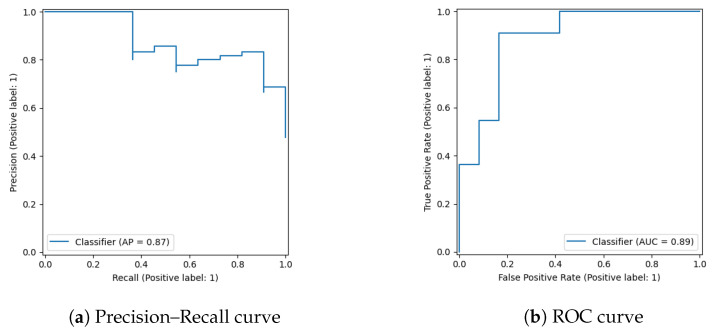
Performance curves of the embedding-based model without multimodal fusion, evaluated on the test set (N = 102). (**a**) Precision–Recall curve with an average precision of 0.87; (**b**) ROC curve with an AUC of 0.89.

**Figure 8:**
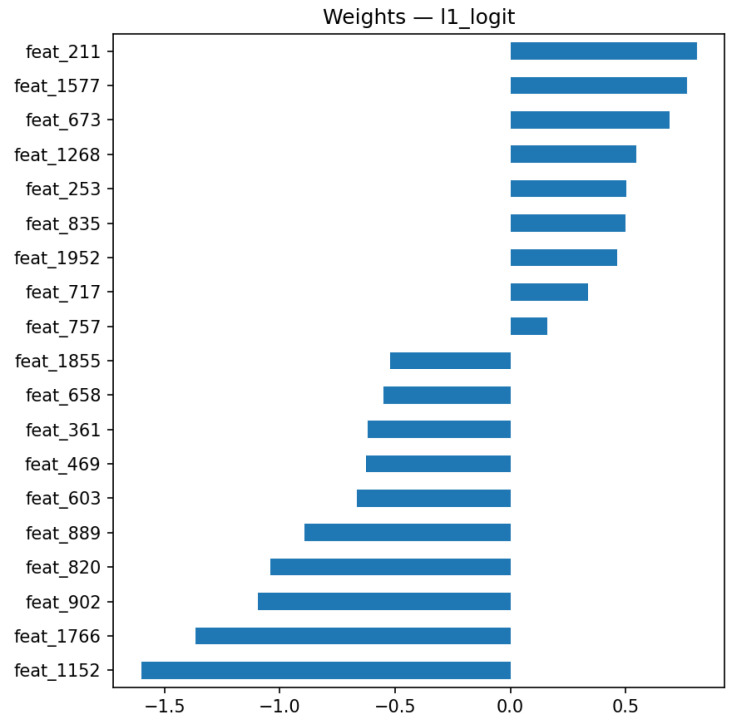
Top 20 feature weights from L1-regularized logistic regression. Interpretable deep features from ResNet embeddings.

**Figure 9:**
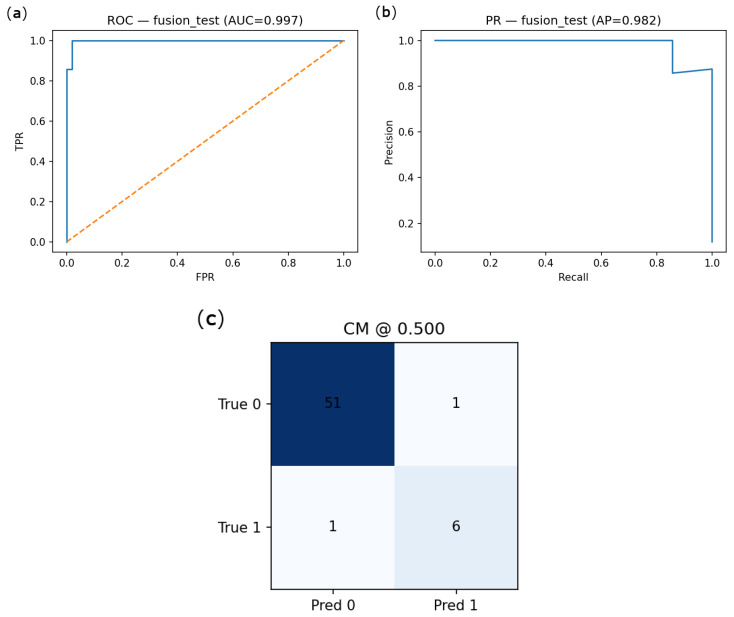
Evaluation of the multimodal fusion model combining deep embeddings and quantitative CT markers on the test set (N = 102). (**a**) ROC curve (AUC = 0.997); (**b**) Precision–Recall curve (AP = 0.982); (**c**) Confusion matrix at decision threshold 0.5.

Primary results are reported on the full, naturally imbalanced cohort using class weighting. Under the 70/15/15 patient-level split, the embedding-based model performance is shown in Figure 7 (Accuracy = 0.83, ROC-AUC = 0.886, PR-AUC = 0.873). Interpretability results are shown in Figure 8. Classification performance for the fusion model and embedding probe is summarized in Table 4 and Figure 9, where uncertainty (95% CI) via 1000-bootstrap is reported.

These results demonstrate a strong discriminatory ability of the model in distinguishing emphysematous lungs from non-pathological ones, even under limited sample size conditions. The high PR-AUC, in particular, indicates robustness in handling class imbalance and clinical relevance of positive detection. As shown in Figure 7, the proposed model demonstrates high performance both in terms of precision–recall and ROC metrics. All metrics are reported as mean ± 95% confidence intervals derived from 1000 bootstrap resamples.

### 4.2. Bootstrapped Evaluation and Feature Selection Strategies

All feature selection procedures were performed exclusively on the training set to prevent information leakage. To investigate the impact of feature selection on the robustness and interpretability of our model, we conducted a comparative analysis of several strategies using logistic regression classifiers trained on (i) the full set of 2048 ResNet embeddings, (ii) top-20 features selected via univariate t-test with FDR correction, (iii) L1-regularized logistic regression (LASSO), and (iv) recursive feature elimination (RFE). All models were evaluated on the held-out test set (n=59) using 1000 bootstrap samples to compute 95% confidence intervals (CI) Table 5. The full-feature logistic model exhibited limited performance and high variability: ROC-AUC 0.695 [0.486; 0.876], PR-AUC 0.382 [0.146; 0.708], F1 0.27 [0.00; 0.57], bACC 0.585 [0.433; 0.761]. This instability highlights the risk of overfitting when using high-dimensional representations without regularization. In contrast, using the top-20 features from the t-test with FDR correction slightly improved classification performance (AUC 0.731), while the L1-regularized model showed a substantial boost: ROC-AUC 0.942, PR-AUC 0.767, F1 0.60, bACC 0.861. The RFE-based model yielded comparable results (ROC-AUC 0.907). These findings demonstrate that robust feature selection is essential for achieving high generalization and interpretable performance without modifying the backbone architecture.

## 5. Discussion

The results indicate that embeddings derived from a domain-adapted ResNet152 provide a compact and informative representation for emphysema classification on low-dose chest CT scans. When combined with statistical analysis and interpretable modeling, these representations achieved strong classification performance (AUC = 0.94) while preserving transparency of the decision process. The relevance of individual embedding dimensions is illustrated in Figure 8, which summarizes their relative contributions and directional effects in the classification model.

Comparative analyses showed that CT-specific pretraining yields more informative features than ImageNet-based or randomly initialized networks, underscoring the importance of task-relevant data for medical image representation learning. In addition, dimensionality reduction methods, including PCA, t-SNE, and UMAP, revealed structure within the embedding space and suggested partial separation between emphysematous and non-emphysematous cases, consistent with the observed classification results.

The proposed workflow—from preprocessing and automatic labeling to feature extraction and evaluation—relies on fully automated and reproducible components, with minimal manual intervention. This design supports scalability and facilitates adaptation to related radiological applications.

Several limitations should be noted. The analysis is restricted to binary classification and depends on LAA-based criteria for label definition. Future studies may extend this framework to regression-based prediction of lung function measures and incorporate spatial feature aggregation to better capture heterogeneity in emphysema patterns.

### 5.1. The Role of Regularization and Feature Selection

The findings highlight the role of appropriate feature selection when modeling high-dimensional embedding spaces. Using all 2048 ResNet-derived features in an unregularized logistic regression led to unstable behavior and limited generalization, reflected by wide bootstrap confidence intervals. By contrast, incorporating regularization or feature selection methods, such as L1-penalized logistic regression (LASSO) or recursive feature elimination (RFE), resulted in more stable models and improved classification performance.

Notably, the L1-regularized logistic model achieved a ROC-AUC of 0.942 and a PR-AUC of 0.767, approaching the performance observed for the multimodal fusion configuration. This suggests that a compact and interpretable model based on a small subset of features can deliver strong performance, making it particularly valuable in clinical settings where interpretability and efficiency are crucial.

### 5.2. Limitations

This study was conducted using a single public dataset (LIDC-IDRI), and no external hold-out cohort was available for validation. Although LIDC-IDRI includes scans acquired with heterogeneous scanners and reconstruction settings, detailed and consistently annotated metadata regarding scanner vendors and reconstruction kernels were not available for all cases. Consequently, stratified subgroup analyses based on acquisition parameters could not be reliably performed.

The reported results should therefore be interpreted as internal validation within a heterogeneous dataset rather than as evidence of cross-center generalizability. External validation on independent, multi-center cohorts with harmonized acquisition metadata remains an important direction for future research.

## 6. Conclusions and Future Directions

In this study, we presented an interpretable methodology for emphysema diagnosis on CT scans using embeddings extracted from a deep convolutional neural network, ResNet152. Rather than directly relying on the network’s predictions as the final output, we implemented an intermediate interpretation layer: the global average pooling features were used as input vectors for simple statistical models. This approach enabled both high classification performance and transparency of computations at the feature level.

According to our results, a linear model trained on the top-20 features selected via Student’s t-test achieved an AUC of 0.94 and AP of 0.87 on a balanced subset. These results indicate strong discriminative power of a small subset of automatically extracted deep features. Examining the coefficients of the logistic regression model highlighted a subset of influential features that may support morphological or pathophysiological interpretation of the underlying imaging patterns.

Rather than centering the analysis on individual performance metrics, this work focuses on the underlying methodological design. We describe a pipeline in which raw CT data are transformed into an interpretable numerical feature space and subsequently analyzed using statistical models. The proposed approach does not rely on end-to-end network training, is reproducible, and can be extended to related applications, such as multiclass classification of emphysema subtypes or estimation of disease severity through regression on LAA%.

### 6.1. Classification Performance: Logits, Embeddings, Fusion

Three classifier configurations were examined: (i) calibrated logits derived from the pretrained CNN, (ii) logistic regression models applied to 2048-dimensional embedding vectors, and (iii) a multimodal fusion approach that combines logits with quantitative CT (QCT) features, including %LAA-950, Perc15, and total lung volume (TLV). All models were trained and evaluated using a stratified train/validation/test split (70/15/15), with patient identifiers tracked to prevent cross-split leakage.

On the validation set, the logits-based model achieved a ROC-AUC of 0.886 and a PR-AUC of 0.511, while the embedding-based probe reached a ROC-AUC of 0.836. Incorporating QCT features in the fusion model resulted in higher performance, with a ROC-AUC of 0.989 and a PR-AUC of 0.898. These results indicate that QCT features provide complementary information to deep embeddings; however, the fusion model was evaluated as an exploratory upper-bound configuration and was not intended to represent a clinically deployable classifier. Fusion performance should therefore be interpreted as an upper-bound estimate within the current labeling scheme. On the test set, fusion performance remained high: ROC-AUC = 0.996, PR-AUC = 0.962, F1 = 0.86, and balanced accuracy = 0.919. Table 4 summarizes these results, along with confusion matrices for interpretability.

We further visualized the test performance of the multimodal fusion model through ROC and PR curves, along with the confusion matrix. The ROC curve (Figure 9a) indicates near-perfect discrimination with an AUC of 0.997, while the PR curve (Figure 9b) highlights excellent performance in identifying emphysema cases, with an average precision of 0.982. As shown in the confusion matrix (Figure 9c), the model achieved both high sensitivity and specificity, misclassifying only one subject in each class.

### 6.2. Scientific and Practical Contributions

- A reproducible framework for constructing interpretable diagnostic models using pretrained convolutional architectures is presented;
- The use of Student’s t-test for selecting informative deep features in combination with linear classifiers is empirically evaluated;
- Coefficients of logistic regression models are used to quantify individual feature contributions, supporting interpretability in a medical context;
- The proposed approach operates without fine-grained annotations or additional labeling, making it applicable to screening scenarios and secondary analysis of existing CT datasets.

### 6.3. Future Work

- Extending the framework to regression tasks for predicting functional respiratory indices (e.g., DLCO, FEV1, FEV1/FVC) from embedding representations;
- Incorporating spatial information by aggregating features across anatomically defined lung regions (upper, middle, and lower lobes);
- Combining deep embeddings with conventional radiomic descriptors to enhance feature diversity;
- Exploring attention-based mechanisms to generate region-level contribution maps;
- Evaluating robustness across different populations and CT acquisition protocols.

Overall, the proposed methodology provides a structured and interpretable approach that is suitable for integration into research pipelines and may support future clinical studies in COPD and emphysema assessment.

### 6.4. Ablation Analysis

An ablation study was performed to examine the relative contribution of individual feature components. Models based exclusively on raw classifier logits exhibited performance close to chance level, whereas the use of embedding-based representations resulted in a clear improvement. Further gains were observed when deep embeddings were combined with quantitative CT (QCT) markers, suggesting that learned features capture information that is complementary to handcrafted descriptors.

Given the limited size of the available cohort, additional feature-selection procedures (such as LASSO or recursive feature elimination) and explicit calibration analyses were not pursued, as these would require further data subdivision and could reduce statistical stability. The ablation results are illustrated by comparing the embedding-based model (Figure 7) with the multimodal fusion model (Figure 9).

## Figures and Tables

**Table 1:** Summary statistics of LAA% across the dataset (N = 497).

Metric	Value
Count	497
Mean	3.31%
Standard Deviation	6.22%
Minimum	0.00%
25th Percentile	0.10%
Median (50th Percentile)	0.73%
75th Percentile	3.38%
Maximum	41.49%

**Table 2:** Top 10 CT scans with the highest LAA% in the dataset.

Patient ID	LAA%	Mean Lung HU	Label
LIDC-IDRI-0309	41.49	−905.0	1
LIDC-IDRI-0140	40.60	−888.0	1
LIDC-IDRI-0105	39.66	−889.0	1
LIDC-IDRI-0422	33.97	−867.3	1
LIDC-IDRI-0196	33.40	−865.0	1
LIDC-IDRI-0467	29.67	−800.1	0
LIDC-IDRI-0041	29.06	−878.7	1
LIDC-IDRI-0297	27.88	−879.8	1
LIDC-IDRI-0457	26.87	−882.5	1
LIDC-IDRI-0450	26.43	−867.3	1

**Table 3:** Descriptive statistics across the training, validation, and test sets.

Split	N	Positives	Negatives	Median %LAA	Mean %LAA	Median TLV (L)	Median Mean HU	Median Perc15 HU
Train	273	35	238	1.39%	3.84%	4.78	−810.3	−903.1
Val	59	8	51	1.14%	3.49%	5.22	−813.2	−902.5
Test	59	7	52	2.06%	4.32%	4.64	−819.0	−908.6

**Table 4:** Classification performance across models on validation and test sets.

Model/Approach	Split	ROC-AUC	PR-AUC	F1	bACC	TN/FP/FN/TP
Logits (TTA + calib)	Val	0.886	0.511	0.55	0.797	43/8/2/6
	Test	0.732	0.332	0.40	0.732	39/13/2/5
Embeddings Probe	Val	0.836	0.507	0.50	0.744	44/7/3/5
	Test	0.766	0.459	0.38	0.690	42/10/3/4
Fusion (logit + QCT)	Val	0.989	0.898	0.84	0.971	48/3/0/8
	Test	0.996	0.962	0.86	0.919	51/1/1/6

**Table 5:** Comparison of feature selection methods on test set (*n* = 59). 95% confidence intervals computed via 1000 bootstrap samples.

Method	ROC-AUC (95% CI)	PR-AUC (95% CI)	F1 (95% CI)	bACC (95% CI)
Logistic regression on all 2048 embeddings	0.695 [0.486; 0.876]	0.382 [0.146; 0.708]	0.27 [0.00; 0.57]	0.585 [0.433; 0.761]
T-test + FDR (top 20)	0.731	0.291	0.23	0.560
L1-logistic (LASSO)	0.942	0.767	0.60	0.861
RFE	0.907	0.569	0.59	0.809

**Figure A1:**
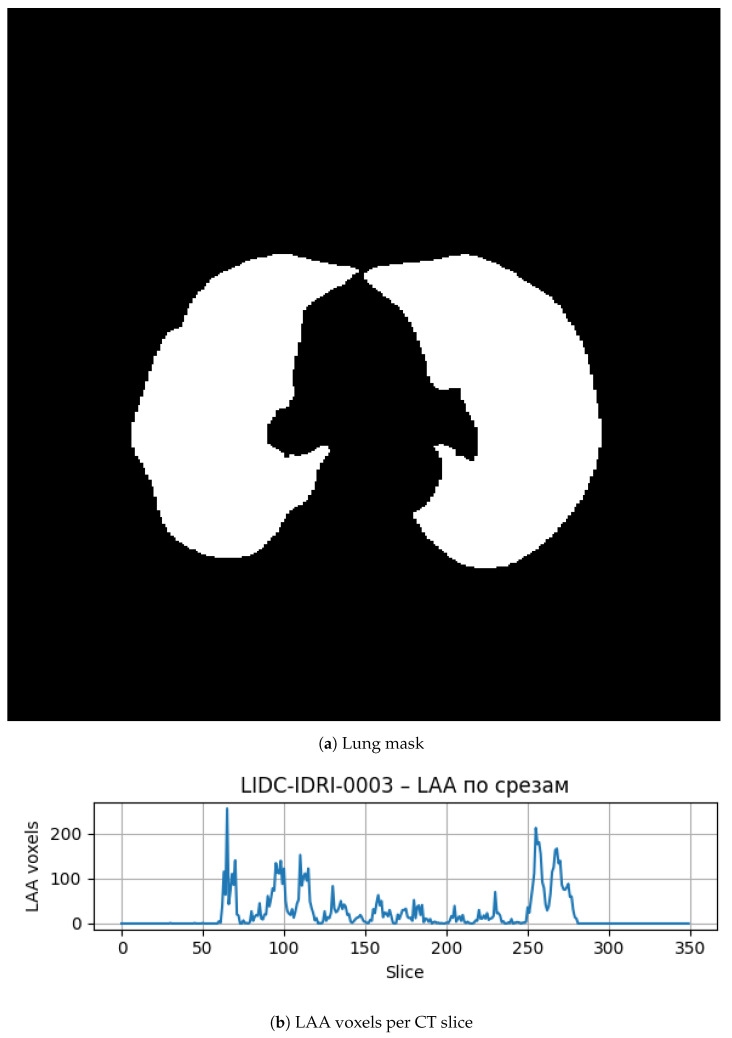
Subject LIDC-IDRI-0003: the segmentation mask covers the lung parenchyma accurately as (**a**) Lung mask. The LAA distribution is sparse and did not exceed the 6% threshold; this subject was labeled as non-emphysematous as (**b**) LAA voxels per CT slice.

**Figure A2:**
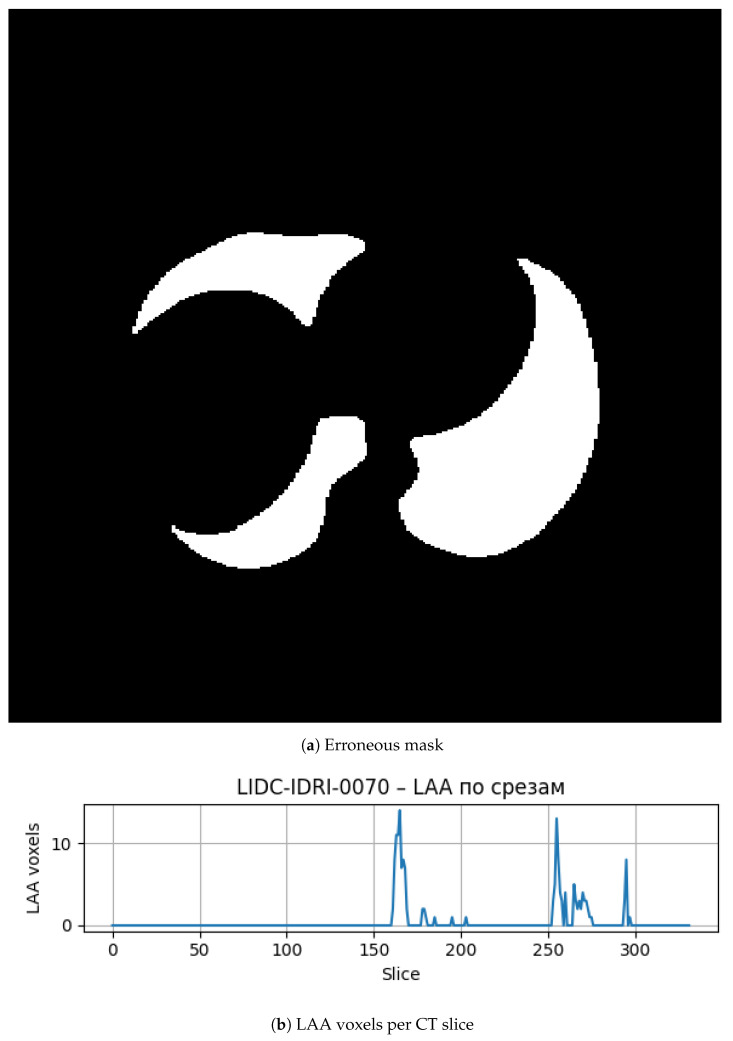
Subject LIDC-IDRI-0070: segmentation failure resulted in incomplete lung coverage as (**a**) Erroneous mask. Despite a low LAA signal, this subject was flagged as low-quality due to mask errors as (**b**) LAA voxels per CT slice.

**Figure A3:**
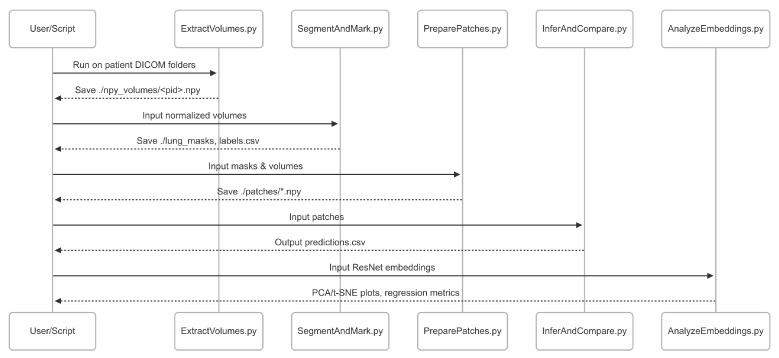
Sequence diagram illustrating the interaction between core scripts in the pipeline for interpretable emphysema diagnosis. The diagram depicts the flow of data between modules: ExtractVolumes.py, SegmentAndMark.py, PreparePatches.py, InferAndCompare.py, and AnalyzeEmbeddings.py, along with intermediate outputs (e.g., masks, predictions, embeddings).

## Data Availability

The data presented in this study are openly available in GitHub at https://github.com/SarsembayevaTalshyn/COPD_AI_in_Med/tree/main (accessed on 12 January 2026).

## Appendix A. Top 20 Patients by LAA Percentage

For further details, we refer the reader to the complete LAA Analysis Report available at: https://drive.google.com/file/d/18v2SkSwNphcNEBQnbVBLl5__rfgjPE5o/view?usp=sharing (acessed on 1 August 2025).

### Appendix A.1. Additional Examples of Segmentation Quality and LAA Distributions

To illustrate segmentation outcomes and LAA-based labeling in more detail, we provide two representative cases below.
